# Disruption of Glycolysis by Nutritional Immunity Activates a Two-Component System That Coordinates a Metabolic and Antihost Response by Staphylococcus aureus

**DOI:** 10.1128/mBio.01321-19

**Published:** 2019-08-06

**Authors:** Paola K. Párraga Solórzano, Jiangwei Yao, Charles O. Rock, Thomas E. Kehl-Fie

**Affiliations:** a Department of Microbiology, University of Illinois at Urbana-Champaign, Urbana, Illinois, USA; b Carl R. Woese Institute for Genomic Biology, University of Illinois at Urbana-Champaign, Urbana, Illinois, USA; c Department of Infectious Diseases, St. Jude Children’s Research Hospital, Memphis, Tennessee, USA; d Departmento de Ciencias de la Vida, Universidad de las Fuerzas Armadas ESPE, Sangolquí, Ecuador; University of Rochester

**Keywords:** ArlRS, *Staphylococcus aureus*, calprotectin, glycolysis, manganese, nutritional immunity

## Abstract

During infection, bacteria use two-component signal transduction systems to sense and adapt to the dynamic host environment. Despite critically contributing to infection, the activating signals of most of these regulators remain unknown. This also applies to the Staphylococcus aureus ArlRS two-component system, which contributes to virulence by coordinating the production of toxins, adhesins, and a metabolic response that enables the bacterium to overcome host-imposed manganese starvation. Restricting the availability of essential transition metals, a strategy known as nutritional immunity, constitutes a critical defense against infection. In this work, expression analysis revealed that manganese starvation imposed by the immune effector calprotectin or by the absence of glycolytic substrates activates ArlRS. Manganese starvation imposed by calprotectin also activated the ArlRS system even when glycolytic substrates were present. A combination of metabolomics, mutational analysis, and metabolic feeding experiments revealed that ArlRS is activated by alterations in metabolic flux occurring in the latter half of the glycolytic pathway. Moreover, calprotectin was found to induce expression of staphylococcal leukocidins in an ArlRS-dependent manner. These studies indicated that ArlRS is a metabolic sensor that allows S. aureus to integrate multiple environmental stresses that alter glycolytic flux to coordinate an antihost response and to adapt to manganese starvation. They also established that the latter half of glycolysis represents a checkpoint to monitor metabolic state in S. aureus. Altogether, these findings contribute to understanding how invading pathogens, such as S. aureus, adapt to the host during infection and suggest the existence of similar mechanisms in other bacterial species.

## Abstract (precis)

Two-component regulatory systems enable bacteria to adapt to changes in their environment during infection by altering gene expression and coordinating antihost responses. Despite the critical role of two-component systems in bacterial survival and pathogenesis, the activating signals for most of these regulators remain unidentified. This is exemplified by ArlRS, a Staphylococcus aureus global regulator that contributes to virulence and to resisting host-mediated restriction of essential nutrients, such as manganese. In this report, we demonstrate that manganese starvation and the absence of glycolytic substrates activate ArlRS. Further investigations revealed that ArlRS is activated when the latter half of glycolysis is disrupted, suggesting that S. aureus monitors flux through the second half of this pathway. Host-imposed manganese starvation also induced the expression of pore-forming toxins in an ArlRS-dependent manner. Cumulatively, this work reveals that ArlRS acts as a sensor that links nutritional status, cellular metabolism, and virulence regulation.

## INTRODUCTION

To successfully establish infection, pathogens must adapt to the dynamic host milieu. This need occurs as host defenses, such as nutrient limitation and antimicrobials, alter local environmental conditions. Among the mechanisms that bacteria use to sense and respond to these changes are the two-component signal transduction systems (TCSs). These regulators reprogram diverse aspects of microbial physiology in response to specific cues by controlling the expression of genes that are essential for bacterial survival, growth, and pathogenesis (1–3). The importance of these systems is exemplified in Staphylococcus aureus, where these regulators coordinate multiple cellular functions, including the expression of virulence determinants (4–8). Given that a wide range of virulence factors can be inhibited by targeting a common regulatory element, such as a TCS, these regulators are attractive targets for antibacterial therapy. To aid the design of novel and effective therapeutics, a better understanding of how TCSs allow bacteria to survive under competitive conditions within the human body is necessary. One of the least understood aspects of TCS signaling is their activation. Indeed, the identity of the signals that stimulate the activity of most of these regulators remains unknown.

S. aureus is a versatile pathogen that can cause devastating disease and that asymptomatically colonizes a third of the human population (9). The importance of adaptation to S. aureus pathogenesis is emphasized by the variety of the infections, ranging from skin and soft tissue infections to more-severe manifestations such as sepsis, endocarditis, and osteomyelitis, that it can cause (10–12). The remarkable capacity of S. aureus to cause disease is largely attributable to its ability to coordinate expression of a copious array of virulence determinants, such as toxins, immune-modulatory factors, and exoenzymes (13). To direct this antihost response, S. aureus uses a plethora of regulators, which include TCSs (14–16). S. aureus has 16 conserved TCSs, including ArlRS, which enable it to respond to its environment (17–21). ArlRS has been shown to contribute to the ability of S. aureus to cause skin infections, sepsis, and endocarditis in several animal models (8, 22–27). The *arl* locus, which encodes the sensor histidine kinase ArlS and the response regulator ArlR, modulates the expression of multiple toxins, exoenzymes, and immune modulators (26). Given that ArlRS also controls expression of various adhesins and surface proteins, it plays a critical role in the regulation of bacterial clumping and adherence (27). Moreover, ArlRS alters metabolism, enabling S. aureus to adapt to disruptions in glycolysis caused by manganese starvation (8). ArlRS regulates these processes directly or via the activity of other staphylococcal regulators, such as Agr, LytSR, MgrA, and Rot (26). Despite the clear importance of the ArlRS system, the molecular signals underlying its activation remain unknown.

During infection, invaders must obtain all of their nutrients from the host. As a countermeasure, the host restricts the availability of these essential nutrients to invaders, a form of defense referred to as nutritional immunity (28–30). The best-characterized example of nutritional immunity is the restriction of essential metals, including manganese (Mn), iron (Fe), and zinc (Zn) (28, 30, 31). As 30% of all proteins are predicted to use a metal (32, 33), this defense is pivotal with respect to the overall host immune response and the outcome of infection. The staphylococcal abscess is the prototypical example of a manganese-withholding response by the host (31, 34). A critical component of this response is the immune effector calprotectin (CP), which is the most abundant cytoplasmic protein in neutrophils and which can be found at foci of infection at concentrations exceeding 1 mg/ml (35, 36). A member of the S100 protein family, CP is a heterodimer comprised of S100A8 and S100A9 (31, 34, 37–39). It contains two metal-binding sites that are capable of sequestering Mn and Zn as well as other transition metals (38, 40–44). In addition to reducing bacterial growth, CP-imposed metal starvation inactivates metal-dependent enzymes and processes, including superoxide dismutases (SODs), which enable bacteria to resist the oxidative burst, and glucose utilization, rendering invading pathogens more sensitive to other host defenses (8, 31, 37, 45). Mice lacking CP have defects in Mn sequestration and are more susceptible to a variety of bacterial and fungal pathogens, including S. aureus, Klebsiella pneumoniae, Acinetobacter baumannii, and Candida albicans (31, 37, 38, 46–49).

Successful pathogens have developed strategies that enable them to overcome host-imposed Mn limitation (28). These adaptations include expression of Mn-sensing regulators and high-affinity Mn transporters, altering cellular metabolism and oxidative stress management (8, 34, 50–63). ArlRS contributes to the ability of S. aureus to resist Mn starvation during infection by promoting amino acid utilization and reducing the cellular demand for manganese that glucose utilization inflicts (8). The necessity of ArlRS for S. aureus to establish invasive disease and resist the restriction of Mn during infection further emphasizes the importance of this regulator (8). Despite the importance of ArlRS, the environmental cues responsible for its activation remain unknown. In this study, we sought to identify the signals that activate ArlRS. Our analysis indicated that disruption of glycolytic flux, resulting either from host-imposed Mn starvation or from growth of S. aureus in the absence of glycolytic substrates, activates this global virulence regulator. The identification of ArlRS as a sensor of glycolytic flux is significant since glucose is the preferred carbon source of S. aureus and multiple other pathogens, and it could explain how invading pathogens adapt to environments where availability of either Mn or glucose is limited. Moreover, analysis of toxin expression revealed that LukED and LukSF-PVL are produced in response to CP in an ArlRS-dependent manner. Overall, these findings reveal the existence of previously unknown links among nutritional immunity, glycolysis, and virulence factor expression.

## RESULTS

### ArlRS is activated upon reduction of intracellular manganese availability.

**FIG 1:**
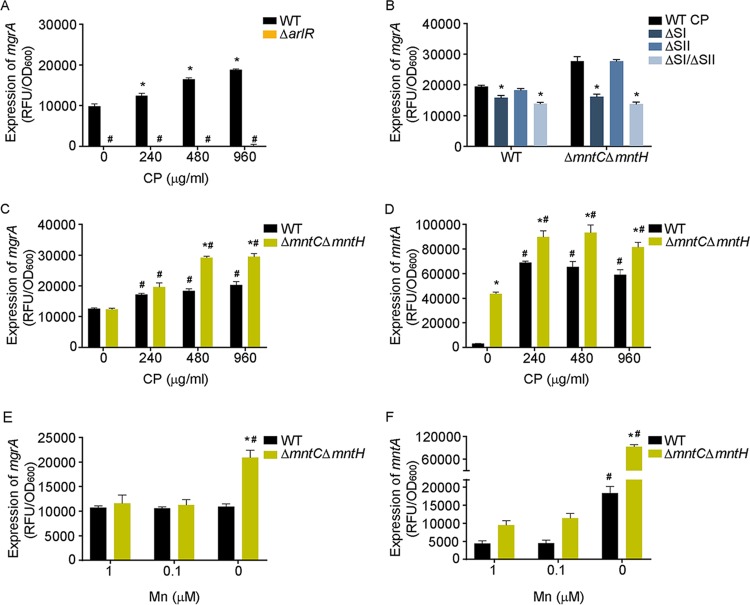
ArlRS is activated in response to reduced levels of intracellular Mn. (A) Wild-type bacteria and a Δ*arlR* mutant were grown in TSB with various concentrations of CP. ArlRS activity was assessed by measuring the activity of the *mgrA* P2 promoter using a YFP-reporter construct at *t* = 6 h. *, *P* ≤ 0.05 (relative to the same strain without CP by two-way analysis of variance [ANOVA] with Dunnett’s multiple-comparison test). #, *P* ≤ 0.05 (relative to wild-type [WT] bacteria at the same CP concentration by two-way ANOVA with Sidak’s multiple-comparison test). RFU, relative fluorescence units. (B) Wild-type S. aureus and a Δ*mntC* Δ*mntH* mutant were grown in TSB with 480 μg/ml WT CP or the ΔSI, ΔSII, or ΔSIΔSII mutant. ArlRS activity was assessed by measuring the activity of the *mgrA* P2 promoter using a YFP-reporter construct at *t* = 9 h. *, *P* ≤ 0.05 (relative to wild-type CP in the same strain by two-way ANOVA with Dunnett’s multiple-comparison test). (C and D) Wild-type bacteria and a Δ*mntC* Δ*mntH* mutant were grown in TSB with increasing concentrations of CP. The activity of the *mgrA* P2 (C) and *mntA* (D) promoters was assessed using YFP-reporter plasmids at *t* = 9 h. *, *P* ≤ 0.05 (relative to wild-type bacteria at the same CP concentration by two-way ANOVA with Sidak’s multiple-comparison test). #, *P* ≤ 0.05 (relative to the same strain in the absence of CP by two-way ANOVA with Dunnett’s multiple-comparison test). (E and F) Wild-type S. aureus and the Δ*mntC* Δ*mntH* mutant were grown in chemically defined medium (CDM) supplemented with various concentrations of manganese, and activity of the *mgrA* P2 (E) and *mntA* (F) promoters was assessed using a YFP-reporter plasmid at *t* = 9 h. *, *P* ≤ 0.05 (relative to wild-type bacteria at the same Mn concentration by two-way ANOVA with Sidak’s multiple-comparison test). #, *P* ≤ 0.05 (relative to the same strain in the presence of 1 μM Mn by two-way ANOVA with Dunnett’s multiple-comparison test). *n* ≥ 3 (all panels). Error bars indicate standard errors of the means (SEM).

ArlRS enhances the ability of S. aureus to resist Mn starvation in culture and during infection (8). Therefore, this TCS could respond to changes in Mn availability. To evaluate this hypothesis, the activity of ArlRS was assessed in the presence and absence of CP. This was accomplished using a transcriptional reporter fusion under the control of the *mgrA* P2 promoter, which is controlled by ArlRS (23). In wild-type bacteria, but not the Δ*arlR* mutant, CP activated the *mgrA* promoter in a dose-dependent manner (Fig. 1A), indicating that metal starvation activates ArlRS. To resolve the issue of whether Mn or Zn sequestration by CP activated ArlRS, we utilized CP variants lacking the Mn/Zn-binding site (ΔSI), the Zn-only-binding site (ΔSII), or both sites (ΔSI/ΔSII) (37, 38, 64–66). ArlRS was activated only in the presence of wild-type CP and the ΔSII variant, indicating that Mn limitation activates the system (Fig. 1B). Altogether, these findings indicate that host-imposed Mn starvation activates ArlRS.

CP reduces both the extracellular and intracellular concentrations of bioavailable Mn. This raises the possibility that ArlRS could be responding to changes in the intracellular or extracellular availability of this metal. In wild-type bacteria, intracellular concentrations of Mn are tied to extracellular abundance. To separate these two compartments, we utilized a S. aureus mutant that lacks the Mn transporters MntABC and MntH (Δ*mntC* Δ*mntH*) (34, 50). Due to its defect in Mn acquisition, the Δ*mntC* Δ*mntH* mutant accumulates less intracellular Mn than wild-type bacteria (50). Hence, if extracellular levels of Mn modulate ArlRS activity, expression of *mgrA* should be equivalent in wild-type bacteria and the Δ*mntC* Δ*mntH* mutant. Conversely, if ArlRS responds to intracellular Mn availability, expression of *mgrA* should be higher in the Δ*mntC* Δ*mntH* mutant than in the wild-type bacteria under conditions of limited Mn availability. In the presence of CP, but not in the presence of metal-replete media, the Δ*mntC* Δ*mntH* mutant exhibited elevated ArlRS activity compared to the wild-type strain (Fig. 1C). Under all of the sets of conditions where ArlRS was activated, increased activity of the promoter for the *mntABC* locus was observed (Fig. 1D). As expression of the MntABC transporter is induced when intracellular manganese levels decrease (34), this indicates that the bacteria are experiencing Mn limitation. As expected in Mn-replete medium, activation of ArlRS was not observed in the Δ*mntC* Δ*mntH* mutant when the ΔSI CP variant was used (Fig. 1B). Cumulatively, these results indicate that ArlRS responded to a reduction in intracellular Mn concentrations. To evaluate whether this was generalizable or specific to CP, we evaluated the activity of ArlRS grown in metal-restricted defined medium supplemented with various concentrations of Mn. Consistent with the CP assays, ArlRS was more active in the Δ*mntC* Δ*mntH* mutant than in wild-type bacteria when no supplemental Mn was added to the medium (Fig. 1E). Surprisingly, activity was observed only in the absence of Mn supplementation and only in the Δ*mntC* Δ*mntH* mutant, although expression of *mntA* in the wild-type and mutant strains indicated that both were experiencing metal limitation (Fig. 1F). While confirming that ArlRS senses reduced intracellular Mn availability, the substantial limitation necessary to activate the system suggests that the system might be responding to disruption of a Mn-dependent process rather than directly sensing Mn availability.

### Pyruvate indirectly activates ArlRS.

**FIG 2:**
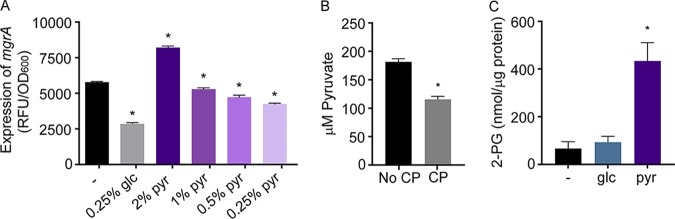
Pyruvate indirectly activates ArlRS. (A) Wild-type S. aureus was grown in yeast complete (YC) medium supplemented with various concentrations of pyruvate (pyr) or glucose (glc). ArlRS activity was assessed by measuring the activity of the *mgrA* P2 promoter using a YFP-reporter plasmid at *t* = 6 h. *, *P* ≤ 0.05 (relative to bacteria grown in the absence of pyruvate by one-way ANOVA with Dunnett’s multiple-comparison test). (B) Wild-type S. aureus was grown in TSB medium in the presence and absence of 240 μg/ml CP, and pyruvate levels were assessed. *, *P* ≤ 0.05 (relative to bacteria grown without CP by unpaired two-tailed *t* test). (C) Wild-type S. aureus was grown in YC medium supplemented with 0.25% glucose or 2% pyruvate, and 2-phosphoglycerate levels were assessed. *, *P* ≤ 0.05 (relative to bacteria grown in YC medium alone by one-way ANOVA with Dunnett’s multiple-comparison test). *n* ≥ 3 (all panels). Error bars indicate SEM.

Previous work demonstrated that ArlRS is a critical component of pyruvate-mediated virulence regulation and suggested that this regulator might directly or indirectly sense pyruvate levels in S. aureus (67). In these studies, an ArlRS-dependent increase in the expression of staphylococcal toxins was observed in yeast complete (YC) medium supplemented with 2% pyruvate compared to unsupplemented medium (67). Mn starvation disrupts the ability of S. aureus to consume glucose (8), leading to the hypothesis that manganese starvation activates ArlRS via pyruvate. As the prior work relied on toxin expression (67), the activity of ArlRS following growth in the presence of pyruvate was initially assessed using the *mgrA* P2 promoter. Consistent with prior results (67), supplementing YC medium with 2% pyruvate, but not at a concentration of 1% or less, increased ArlRS activity (Fig. 2A). Building on this observation, pyruvate accumulation was assessed in S. aureus grown in the presence of concentrations of CP that activate ArlRS. These assays revealed that CP reduced intracellular pyruvate levels (Fig. 2B), suggesting that pyruvate indirectly activates ArlRS.

### The absence of glycolytic substrates activates ArlRS.

**FIG 3:**
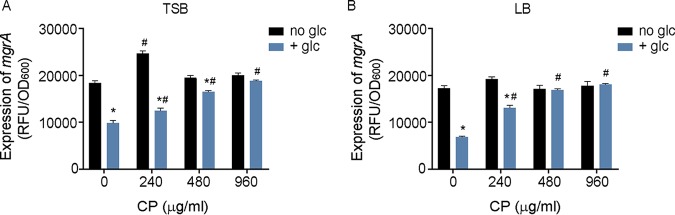
The absence of glucose and manganese starvation activate ArlRS. Wild-type S. aureus was grown in TSB (A) or LB (B) supplemented with 0.25% glucose (+glc) or without glucose (no glc) as indicated. ArlRS activity was assessed by measuring the activity of the *mgrA* P2 promoter using a YFP-reporter plasmid at *t* = 6 h. *, *P* ≤ 0.05 (relative to bacteria grown without glucose at the same CP concentration by two-way ANOVA with Sidak’s multiple-comparison test). #, *P* ≤ 0.05 (relative to bacteria grown in the same medium without CP by two-way ANOVA with Dunnett’s multiple-comparison test). *n* ≥ 3. Error bars indicate SEM.

The addition of 2% pyruvate increases 2-phosphoglycerate (2-PG) levels in S. aureus (Fig. 2C) and alters the expression of metabolic genes (67). Those observations and additional prior results (8) suggest a link between ArlRS activity and glycolysis. To test this idea, the impact of glucose on ArlRS activity was evaluated. Growth of S. aureus in tryptic soy broth (TSB) without glucose and in TSB with glucose (0.25%) revealed that ArlRS activity was ∼2 times higher in the cells grown in the absence of glucose than in those cultivated in its presence (Fig. 3A). Consistent with these observations, a similar change in ArlRS activity occurred when S. aureus was grown in Luria broth (LB) and LB supplemented with 0.25% glucose (Fig. 3B). The addition of 0.25% glucose to YC media also reduced the activity of ArlRS (Fig. 2A). The latter observation also indicates that the addition of 2% pyruvate to this medium leads to further activation of a system that is already on. Together, these observations indicate that the increased activity of ArlRS was due to the absence of glucose and not to the presence of or absence of other components of these media.

**FIG 4:**
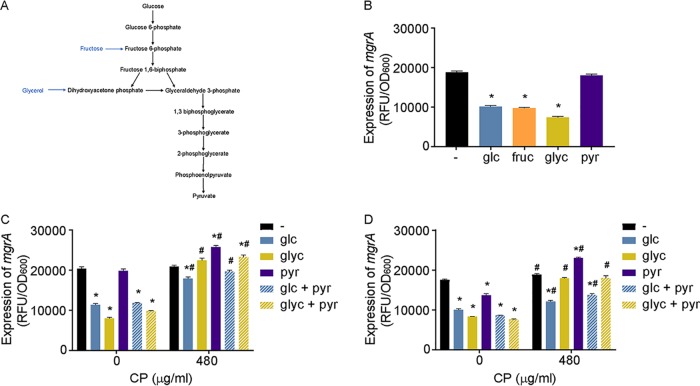
Disruption of the latter half of glycolysis activates ArlRS. (A) Simplified representation of glycolysis indicating where fructose and glycerol feed into the pathway. (B) Wild-type S. aureus Newman was grown in TSB without glucose and supplemented with carbon-balanced concentrations of glucose (glc), fructose (fru), glycerol (glyc), or pyruvate (pyr). (B to D) ArlRS activity was assessed by measuring the activity of the *mgrA* P2 promoter using a YFP-reporter plasmid at *t* = 6 h. (B) *, *P* ≤ 0.05 (relative to medium without glucose by one-way ANOVA with Dunnett’s multiple-comparison test). (C and D) Wild-type S. aureus Newman (C) and USA300 JE2 (D) were grown in TSB without glucose and without supplementation (−) or supplemented with carbon-balanced concentrations of glucose (glc), glycerol (glyc), and/or pyruvate (pyr) in the absence and presence of 480 μg/ml CP. *, *P* ≤ 0.05 (relative to growth in medium without glucose at the same CP concentration by two-way ANOVA with Dunnett’s multiple-comparison test). #, *P* ≤ 0.05 (relative to growth in the same carbon source without CP by two-way ANOVA with Sidak’s multiple-comparison test). *n* ≥ 3 (panels B to D). Error bars indicate SEM.

To evaluate the possibility that ArlRS was directly sensing the presence of glucose, we assessed the impact that other carbon sources had on its activity. Similarly to the results seen with glucose, ArlRS activation was repressed by fructose and glycerol, which feed into glycolysis at the level of fructose-6-phosphate and dihydroxyacetone phosphate (Fig. 4A), respectively (Fig. 4B). These observations indicate that lack of flux through the first half of glycolysis did not impact ArlRS activity and that the system was not sensing glucose directly. On the other hand, S. aureus grown in the presence of pyruvate (0.25%) exhibited increased ArlRS activity compared to that observed in cells grown in the presence of glucose (Fig. 4B). The elevated ArlRS activity seen under these conditions was presumably due to reduced glycolytic flux, as ArlRS activity in pyruvate-containing medium was repressed by reconstitution of glycolytic activity with either glucose or glycerol (Fig. 4C). Cumulatively, these results suggest that reduced glycolytic flux activates ArlRS.

### Manganese limitation activates ArlRS regardless of the glycolytic carbon source present.

To determine if the activities of the two stimuli that modulate ArlRS activation are interconnected, the activity of this TCS was assessed in S. aureus grown in TSB or LB with or without glucose in the presence of various concentrations of CP. Treatment with CP increased ArlRS activity in medium containing glucose in a dose-dependent manner (Fig. 3). However, CP treatment did not increase ArlRS activity when the bacteria were grown in the absence of glucose. CP also increased the activity of ArlRS when glycerol was provided as a carbon source (Fig. 4C). When grown in the presence of 0.25% pyruvate, a condition that results in activation comparable to that seen in the absence of glucose or glycerol (Fig. 4B), only minimal induction was observed when CP was added to the growth medium (Fig. 4C). Similar results were observed in the presence and absence of CP with the methicillin-resistant USA300 JE2 strain (Fig. 4D). Together with the observation that Mn starvation prevents S. aureus from using glucose as an energy source (8), these data suggest that disruption of glycolysis by CP activates ArlRS.

### Disruption of the latter half of glycolysis activates ArlRS.

**FIG 5:**
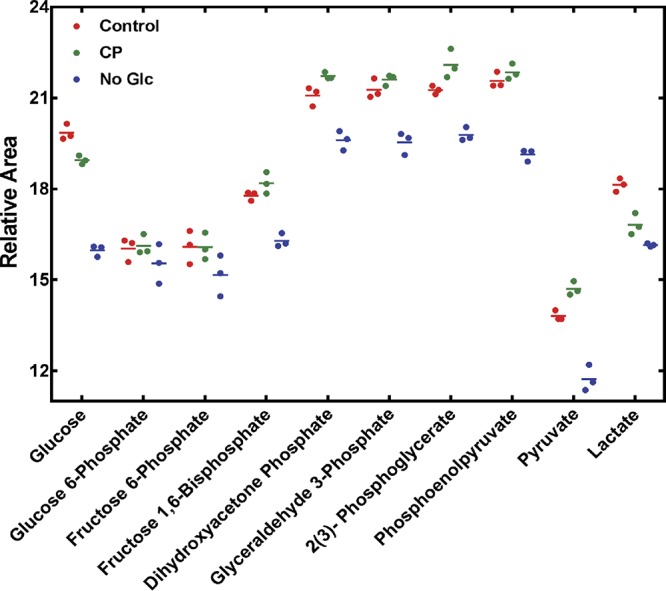
Relative levels of glycolytic intermediates in cells treated with CP or deprived of glucose. S. aureus was grown either in the presence of 120 μg/ml CP or without glucose, and the relative levels of glycolytic intermediates were measured using mass spectrometry as described in Materials and Methods. The level of each metabolite was normalized to the total ion current of the 86 metabolites detected. Each dot represents an independent measurement, and means are indicated by horizontal lines.

To understand how disruption of glycolytic flux activates ArlRS, the levels of glycolytic intermediates in bacteria grown in the absence of glucose were compared to the levels seen with those grown in the presence of glucose but treated with CP, using mass spectrometry. Bacterial cells grown in the absence of glucose contained lower levels of intracellular glucose, fructose 1,6-biphosphate, dihydroxyacetone phosphate, glyceraldehyde 3-phosphate, 2(3)-phosphoglycerate (the two forms were not differentiated by mass spectroscopy), phosphoenolpyruvate, and pyruvate than cells grown in the presence of this stimulus (Fig. 5). CP alters normal glycolytic activity, as it results in a substantial decrease in the levels of intracellular glucose and lactate coupled with an increase in the levels of dihydroxyacetone phosphate and 2(3)-phosphoglycerate (Fig. 5). These data are consistent with CP disrupting glycolytic flux as previously suggested (8). However, no common changes in glycolytic intermediates were observed between bacteria grown in the presence of CP and those grown in the absence of glucose when compared to bacteria grown in metal-replete medium containing glucose.

While metabolomics failed to identify a metabolite change that connected CP treatment and glucose withdrawal, the mass spectrometry approach utilized did not detect 1,3-bisphosphoglycerate and cannot distinguish 3-phosphoglycerate from 2-phosphoglycerate. 2-PG levels were assessed using enzymatic analysis to determine if a reduction in 2-PG correlated with activation of ArlRS. The abundance of this intermediate was assessed in S. aureus grown under conditions that activate this TCS, including 2% pyruvate-containing YC medium, TSB without glucose, and TSB with CP. Enzymatic analysis revealed that, although cells grown in the absence of glucose accumulated less 2-PG than cells grown in its presence, S. aureus grown in the presence of both CP and 2% pyruvate contained elevated 2-PG levels compared to bacteria grown in the absence of these stimuli (Fig. 2C; see also 6A and B). The divergent concentrations of 2-PG observed in these three conditions suggest that changes in this metabolite are not responsible for activating ArlRS.

**FIG 6:**
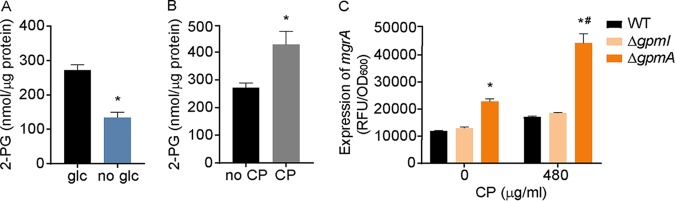
Accumulation of 3-PG or of other upstream intermediates activates ArlRS. (A and B) Wild-type S. aureus was grown in TSB without glucose (no glc) supplemented with 0.25% glucose (glc) (A) or in glucose-containing TSB in the absence and presence of 480 μg/ml CP (B), and 2-phosphoglycerate levels were assessed. *, *P* ≤ 0.05 (relative to bacteria grown in the absence of either glucose [A] or CP [B] by unpaired two-tailed *t* test). (C) Wild-type, Δ*gpmI*, and Δ*gpmA* S. aureus were grown in TSB in the absence and presence of 480 μg/ml CP. ArlRS activity was assessed by measuring the activity of the *mgrA* P2 promoter using a YFP-reporter plasmid at *t* = 6 h. *, *P* ≤ 0.05 (relative to wild-type bacteria at the same CP concentration by two-way ANOVA with Dunnett’s multiple-comparison test). #, *P* ≤ 0.05 (relative to the same strain in the absence of CP by two-way ANOVA with Sidak’s multiple-comparison test). *n* ≥ 3 (panels A to C). Error bars indicate SEM.

The increase in 2-PG levels in the presence of pyruvate and CP led us to evaluate if accumulation rather than depletion of an intermediate activated ArlRS. In light of the mass spectrometry results, the possibility that accumulation of either 1,3-bisphosphoglycerate (1,3-BPG) or 3-phosphoglycerate (3-PG) might activate ArlRS was considered. S. aureus has two phosphoglycerate mutases, namely, GpmA and GpmI. GpmI requires Mn for activity, while GpmA does not (68, 69). We determined whether interfering with the conversion of 3-PG to 2-PG affects ArlRS activity by deleting each of these genes and growing the mutants in the presence of CP. In the presence of CP, loss of GpmA increased ArlRS activity compared to the levels seen with either wild-type bacteria or the Δ*gpmI* mutant (Fig. 6C). As the Δ*gpmA* mutant must use the Mn-dependent GpmI, which should be inhibited by CP, this indicates that disrupting phosphoglycerate mutase activity increases the activity of ArlRS. We also observed that ArlRS activity was modestly higher in the Δ*gpmA* mutant in Mn-replete medium. This observation suggests the GpmA is the main enzyme catalyzing this step of glycolysis when Mn is freely available. Together, these observations suggest that accumulation of either 3-PG or 1,3-bisphosphoglycerate could activate ArlRS.

### CP induces ArlRS-dependent expression of pore-forming leukocidins.

**FIG 7:**
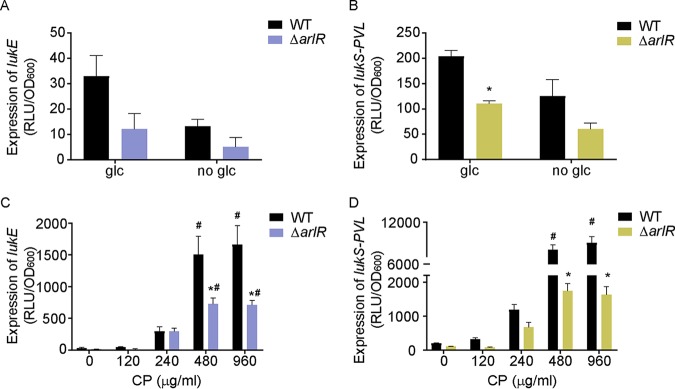
CP induces ArlRS-dependent expression of pore-forming leukocidins. (A to D) Wild-type and Δ*arlR* S. aureus USA300 JE2 were grown in TSB without glucose (no glc), supplemented with 0.25% glucose (glc) (A and B) or glucose-containing TSB in the presence of increasing concentrations of CP (C and D). Leukocidin expression was assessed by measuring the activity of the *lukE* (A and C) and *lukS-*PVL (B and D) promoters using a Lux-reporter plasmid at *t* = 6 h. (A to D) *, *P* ≤ 0.05 (relative to wild-type bacteria under each condition by two-way ANOVA with Sidak’s multiple-comparison test). (C and D) #, *P* ≤ 0.05 (relative to the same strain in the absence of CP by two-way ANOVA with Dunnett’s multiple-comparison test). *n* ≥ 3 (panels A to D). Error bars indicate SEM.

ArlRS coordinates the expression of virulence determinants, including the leukocidins LukED and LukSF-PVL (8, 22, 24, 26). Therefore, we investigated the impact that availability of glucose and Mn would have on the expression of these two leukocidins. Surprisingly, no induction of either toxin in either wild-type bacteria or a Δ*arlR* mutant was observed in glucose-free medium relative to medium containing glucose (Fig. 7A and B). However, in the presence of CP, the expression levels of *lukED* and *lukSF-PVL* increased ∼50-fold and ∼48-fold, respectively, in an ArlRS-dependent manner (Fig. 7C and D). Collectively, these observations indicate that ArlRS contributes to the expression of staphylococcal virulence factors in response to nutritional immunity.

## DISCUSSION

**FIG 8:**
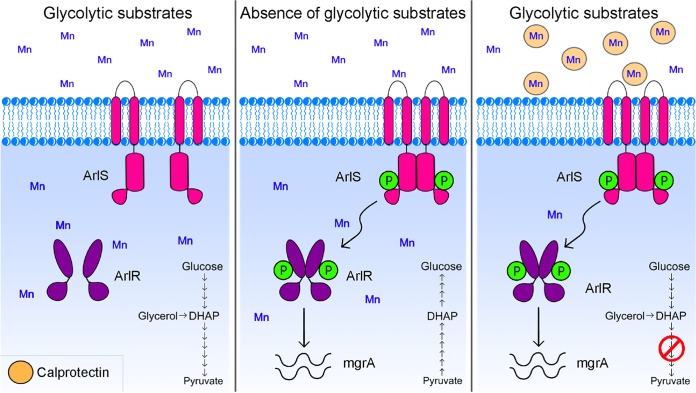
Model of ArlRS activation. When glycolytic carbon sources (such as glucose and glycerol) and manganese are abundant in the host environment, ArlRS remains inactive. However, in an environment poor in glycolytic carbon sources or under conditions of host-imposed manganese starvation, both stimuli (which alter flux in the latter half of glycolysis) elicit the activation of ArlRS. Activation of this TCS allows S. aureus to coordinate an antihost response consisting of the production of virulence factors such as leukocidins and results in reprogramming of staphylococcal metabolism in response to nutritional immunity.

TCSs play an important role in allowing pathogens to adapt to the hostile host environment, as they enable bacteria to perceive environmental changes and reprogram their physiology in response to stress (1–3). Despite the importance of TCSs in bacterial pathogenesis, the identity of the activating signals for most of these regulators remains unclear. The current investigations revealed that the global virulence regulator ArlRS responds to alterations in metabolic flux occurring in the latter half of the glycolytic pathway (Fig. 8). This represents a new regulatory checkpoint for glycolysis. ArlRS is required for maximal toxin expression in response to CP-imposed disruption of glycolysis, and this sensor enables the integration of multiple inputs to coordinate an antihost response. Together, these findings highlight previously unappreciated connections among nutritional immunity, metabolism, and virulence and broaden our understanding of the signals utilized by invading pathogens to thrive within the host.

ArlRS was recently suggested to respond to pyruvate availability (67). However, the current investigations suggested that activation of ArlRS by pyruvate is indirect. The current studies also revealed that ArlRS responds to intracellular Mn availability. Notably, both CP and 2% pyruvate either disrupt glycolysis or alter the expression of glycolytic enzymes or both (8, 67). While the specific step in glycolysis that is disrupted by CP is unknown, excess pyruvate appears to drive staphylococcal metabolism toward gluconeogenesis (67). The latter idea is supported by the current observation that 2% pyruvate increased cellular levels of 2-PG. ArlRS is also activated by the absence of glycolytic intermediates that enter the pathway above 1,3-BPG, which would also trigger gluconeogenesis. Differing from the results seen with CP and 2% pyruvate, 2-PG levels decrease in the absence of glucose, suggesting that an upstream metabolite rather than 2-PG activates ArlRS. This idea is supported by the observation that ablation of staphylococcal phosphoglycerate mutase activity, which presumptively leads to the accumulation of 3-PG and other upstream metabolites, activates ArlRS. Combined with the results of metabolomics analyses, which failed to detect common changes in other glycolytic intermediates, these observations suggest that elevation of the level of 3-PG or 1,3-BPG results in ArlRS activation.

In S. aureus and other pathogens, metabolism and virulence factor expression are intertwined (70–74). In Gram-positive bacteria, the carbon catabolite repressor CcpA is important for the regulation of streptococcal capsular biosynthesis and the expression of a variety of toxins in organisms such as Clostridium difficile, Bacillus anthracis, and S. aureus (72, 75). Similarly, the branched-chain amino acid sensor CodY acts as a toxin gene regulator in S. aureus, C. difficile, B. anthracis, and Listeria monocytogenes (72, 75, 76). Rex, a redox-dependent transcriptional repressor, negatively regulates expression of genes involved in respiration and promotes toxin expression (72, 74, 75). The current investigations revealed that ArlRS responds to the abundance of intermediates formed in the second half of glycolysis, a novel node for regulatory control, and coordinates the expression of virulence factors such as adhesins and toxins (27, 67). Intriguingly, our observations suggest that ArlRS is part of a layered response. Both the absence of glycolytic substrates and the absence of CP activate the *mgrA* promoter, which is associated with controlling staphylococcal adherence and clumping (23). However, expression of LukED and LukSF-PVL changes only in response to CP, indicating that a second signal is necessary. This idea is further supported by the observation that in YC medium, ArlRS activation by pyruvate is also sufficient to induce staphylococcal toxin expression (67). CP enhances the activity of the SaeRS TCS, which regulates S. aureus toxin expression (21, 77, 78), suggesting that this system may provide the second signal. Importantly, the induction of LukED and LukSF-PVL in response to CP is not simply due to activation of the SaeRS system, as loss of ArlR ablates CP-mediated induction of the toxins. Both LukED and LukSF-PVL target neutrophils, which are the primary source of CP during staphylococcal infection (79). While increased toxin expression is potentially beneficial in the presence of neutrophils, it is difficult to envision how this would benefit S. aureus in the absence of glycolytic substrates. Thus, it is tempting to speculate that the multiple layers of regulation enable S. aureus to fine-tune a response triggered by a system that monitors a pathway that can be disrupted by multiple stressors.

Glycolysis is tightly regulated by the environment and flux through the pathway. The mechanisms by which both Gram-positive and Gram-negative organisms sense flux through the first half of glycolysis are well established (72, 80). In Gram-positive bacteria, including S. aureus, fructose bisphosphatase (FBP) regulates glycolysis by two mechanisms. First, FBP promotes the import of glucose and other sugars via the CcpA/Hpr pair, allowing bacteria to selectively use glycolytic substrates when diverse carbon sources are present (81, 82). Second, FBP allosterically stimulates pyruvate kinase activity, enabling S. aureus and other bacteria to ensure that the glucose brought into the cell is flowing through the first half of glycolysis (83, 84). And yet bacteria encounter stresses (including Mn limitation; copper, nickel, and fluoride intoxication; and nitrosative damage) that disrupt glycolysis after the import and conversion of glucose to FBP but before the production of pyruvate (8, 85–88). The current work revealed that the activity of ArlRS changes in response to disruptions in the second half of glycolysis triggered by host-imposed Mn limitation. This enables S. aureus to shift away from carbohydrate catabolism to amino acid degradation, enabling the bacterium to circumvent the impact of nutritional immunity (8). In other bacteria, including Escherichia coli, Salmonella enterica serovar Typhimurium, Bacillus stearothermophilus, and Bacillus subtilis, a step after the formation of FBP is known to be or is likely to be manganese dependent (89–93). Similarly to S. aureus, Streptococcus pneumoniae shifts metabolism away from glucose utilization under conditions of Mn starvation (62). Additionally, the ResDE two-component system in Bacillus subtilis has been suggested to respond to 1,3-bisphosphoglycerate levels enabling anaerobic growth (94). Although no obvious gene targets are shared between ArlRS and ResDE, both respond to disruptions in the latter half of glycolysis and enable adaptation to environmental stress (26, 95, 96). Together, these observations suggest that monitoring the second half of the glycolytic pathway may be a common strategy employed by bacteria.

Adaptation to a changing environment is critical for survival, and it is possible for multiple stresses to disrupt the same metabolic pathway. Sensing intracellular metabolic flux enables bacteria to monitor disruption of critical metabolic pathways, broadening their ability to respond to stressors. The current investigation established that ArlRS is activated by decreased sugar availability and nutritional immunity, both of which alter flux in the latter half of glycolysis, identifying this metabolic segment as a new regulatory node. Further investigations will increase our understanding of how S. aureus and potentially other pathogens leverage changes in the latter half of glycolysis to coordinate their response to nutrient abundance and the immune response.

## MATERIALS AND METHODS

### Bacterial strains and cloning.

S. aureus strains were grown at 37°C in tryptic soy broth (TSB) on a roller drum or on tryptic soy agar (TSA) plates for performing routine culturing or for genetic manipulation. E. coli strains were routinely cultivated at 37°C in Luria broth (LB) with shaking or on Luria agar plates. As needed for plasmid maintenance in E. coli and S. aureus, 100 μg/ml of ampicillin and 10 μg/ml of chloramphenicol were added to the growth medium, respectively. Both bacterial species were stored at −80°C in growth medium containing 30% glycerol.

S. aureus Newman and its derivatives were used for all experiments, unless otherwise indicated. For experiments using USA300 JE2 and the derivative USA300 JE2 *arlR*::erm, strains were obtained from the Nebraska library (97). The Δ*arlR* and Δ*arlR* Δ*mntC* Δ*mntH* mutants were generated by the use of phage transducing the *arlR*::erm allele from USA300 JE2 into the wild-type and Δ*mntC* Δ*mntH* backgrounds (Φ85 phage). For fluorescent reporters, the upstream promoter of *mgrA* P2 and the promoter of *mntA* were cloned into yellow fluorescent protein (YFP)-containing vector pAH5 (98) using the indicated primers (see Table S1 in the supplemental material). Plasmids were electroporated into S. aureus RN4220 (99) before being transferred into final recipient strains, as previously described (100). All constructs were verified by sequencing. The hemolytic activity of all staphylococcal strains was confirmed by plating on blood agar plates. See Tables S2 and S3 for the full lists of strains and plasmids utilized in this study.

10.1128/mBio.01321-19.1 TABLE S1 Primers used in this study. Download Table S1, DOCX file, 0.01 MB. Copyright © 2019 Párraga Solórzano et al. 2019 Párraga Solórzano et al. This content is distributed under the terms of the Creative Commons Attribution 4.0 International license.

10.1128/mBio.01321-19.2 TABLE S2 Staphylococcus aureus strains used in this study. Download Table S2, DOCX file, 0.02 MB. Copyright © 2019 Párraga Solórzano et al. 2019 Párraga Solórzano et al. This content is distributed under the terms of the Creative Commons Attribution 4.0 International license.

10.1128/mBio.01321-19.3 TABLE S3 Plasmids used in this study. Download Table S3, DOCX file, 0.02 MB. Copyright © 2019 Párraga Solórzano et al. 2019 Párraga Solórzano et al. This content is distributed under the terms of the Creative Commons Attribution 4.0 International license.

### ArlRS activity reporter assays.

For assays assessing ArlRS activity in the presence of CP, bacteria were grown as previously described with minor modifications (8, 37, 38, 101). Briefly, overnight cultures were back-diluted 1:100 in 96-well round-bottom plates containing 100 μl/well of growth medium, consisting of 38% of the indicated complex medium and 62% calprotectin buffer (20 mM Tris [pH 7.5], 100 mM NaCl, 3 mM CaCl_2_, 10 mM β-mercaptoethanol). This medium was supplemented with 1 μM MnCl_2_ and 1 μM ZnSO_4_. All cultures were incubated at 37°C with shaking at 180 rpm. Growth was assessed by measuring optical density at 600 nm (OD_600_). Expression of *mgrA* and *mntA* was determined by measuring yellow fluorescence (excitation and emission wavelengths of 505 nm and 535 nm, respectively) and normalizing to OD_600_, as previously described (63, 101, 102). Expression of *lukE* and *lukS-*PVL was determined by measuring luminescence and normalizing to OD_600_. When indicated, equimolar concentrations of carbon from glucose (5.34 mM), fructose (5.34 mM), glycerol (10.68 mM), or sodium pyruvate (10.68 mM) were provided.

For assays assessing ArlRS activation in the presence of different concentrations of manganese, S. aureus was grown overnight in 5 ml of NRPMI (Chelex-treated RPMI) (34) supplemented with 1 mM MgCl_2_, 100 μM CaCl_2_, 1 μM ZnSO_4_ and 1 μM FeCl_2_. The overnight cultures were back-diluted 1:100 in 96-well round-bottom plates containing 100 μl/well of growth medium, consisting of 38% chemically defined medium (2.6×) and 62% calprotectin buffer (20 mM Tris [pH 7.5], 100 mM NaCl, 1 mM CaCl_2_, 10 mM β-mercaptoethanol) (8). The defined medium (2.6×) consisted of 0.5 g/liter NaCl, 1.0 g/liter NH_4_Cl, 2 g/liter KH_2_PO_4_, 7 g/liter Na_2_HPO_4_, 0.228 μg/liter biotin, 0.228 mg/liter nicotinic acid, 0.228 mg/liter pyridoxine-HCl, 0.228 mg/liter thiamine-HCl, 0.114 mg/liter riboflavin, 0.684 mg/liter calcium pantothenate, 0.104 g/liter phenylalanine, 0.078 g/liter isoleucine, 0.130 g/liter tyrosine, 0.053 g/liter cysteine, 0.260 g/liter glutamic acid, 0.026 g/liter lysine, 0.182 g/liter methionine, 0.078 g/liter histidine, 0.026 g/liter tryptophan, 0.234 g/liter leucine, 0.234 g/liter aspartic acid, 0.182 g/liter arginine, 0.078 g/liter serine, 0.156 g/liter alanine, 0.078 g/liter threonine, 0.130 g/liter glycine, 0.208 g/liter valine, and 0.026 g/liter proline. Glucose (1.3%) was provided as the carbon source. This medium was supplemented with 2.3 mM MgSO_4_, 1 μM ZnSO_4_, 1 μM FeSO_4_, and the indicated concentration of MnCl_2_.

For assessing ArlRS activation in YC medium, overnight cultures grown in YC medium were back-diluted 1:100 in 96-well round-bottom plates containing 100 μl/well of YC medium supplemented with glucose (5.34 mM) or with 0.25%, 0.5%, 1%, or 2% sodium pyruvate or with both, as indicated.

### Pyruvate assays.

Overnight cultures were back-diluted 1:100 in 96-well round-bottom plates containing 100 μl/well of growth medium consisting of 38% TSB (either with or without glucose) and 62% calprotectin buffer (20 mM Tris [pH 7.5], 100 mM NaCl, 3 mM CaCl_2_, 10 mM β-mercaptoethanol) and were grown in the absence or presence of 240 μg/ml CP. Bacteria were harvested during logarithmic-phase growth at similar optical densities (OD_600_ = 0.2 to 0.25). Bacterial culture (∼60 ml) was collected in a Millipore S-Pak membrane filter, washed twice with 0.5% NaCl, and stored at −80°C in 60% (vol/vol) high-performance liquid chromatography (HPLC)-grade ethanol. Prior to assay of pyruvate, cells were thawed, washed from the filter, and then lysed by mechanical disruption using a FastPrep-24 homogenizer (MP Biomedicals) for 45 s at 6.5 m/s. Cell lysates were clarified by centrifugation at 10,000 × *g* for 5 min at 4°C. The supernatants were collected and vacuum dried. The dehydrated pellets were resuspended in 100 μl of HPLC-grade water, and then pyruvate levels were measured using an EnzyChrom pyruvate assay kit (BioAssay Systems) as indicated by the manufacturer.

### 2-Phosphoglycerate assays.

For 2-phosphoglycerate determination, bacteria were grown and processed as previously described for pyruvate assays using either growth medium consisting of 38% TSB (either with or without glucose) and 62% calprotectin buffer in the absence or presence of 240 μg/ml CP or growth medium consisting of YC medium supplemented with 0.25% glucose or 2% sodium pyruvate or both, as indicated. The dehydrated pellets obtained after processing were resuspended in 100 μl of 2-PG assay buffer, and then 2-phosphoglycerate levels and total protein concentrations were measured using a 2-phosphoglycerate colorimetric/fluorometric assay kit (BioVision) and a Pierce bicinchoninic acid (BCA) protein assay kit, respectively.

### Metabolomics analysis.

For metabolomics analysis, bacteria were grown and processed as described for the pyruvate assays using either growth medium consisting of 38% TSB (either with or without glucose) or 62% calprotectin buffer in the absence or presence of 120 μg/ml CP and were harvested during logarithmic-phase growth at an OD_600_ of 0.1 to 0.15. The dehydrated pellets were resuspended in 80% methanol. Metabolites were analyzed using a Shimadzu Prominence Ultra Fast Liquid Chromatograph (UFLC) attached to a Sciex QTrap 4500 system equipped with a Turbo V ion source (Sciex). Samples (5 μl) were injected into an XSelect HSS C_18_ column (2.5-μm pore size, 3.0 by 150 mm) using a flow rate of 0.3 ml/min. Solvent A contained 100 mM ammonium formate (pH 5.0), 2% acetonitrile, and 0.1% t-butanol. Solvent B was composed of 95% acetonitrile, 50 mM ammonium formate (pH 6.3), and 0.1% t-butanol. The HPLC program was as follows: starting solvent mixture of 0% solvent B, 0-to-2-min isocratic with 0% solvent B; 2-to-12-min linear gradient to 5% solvent B; 12-to-17-min linear gradient to 90% solvent B; 17-to-25-min isocratic with 90% solvent B; 25-to-27-min linear gradient to 0% solvent B; 27-to-30-min isocratic with 0% solvent B. The Sciex QTrap 4500 system was operated in the negative mode, and the ion source parameters were as follows: ion spray voltage, −4,500 V; curtain gas pressure, 40 lb/in^2^; temperature, 500°C; collision gas setting, high; ion source gas 1 pressure, 50 lb/in^2^; ion source gas 2 pressure, 50 lb/in^2^. Multiple-reaction monitoring (MRM) precursor and product ions were derived as described previously by Bajad et al. (103). The system was controlled by the use of Analyst software and analyzed with MultiQuant 3.0.2 software (Sciex, Inc.). The relative levels of individual metabolite were determined by normalizing each signal to the total current sum of ions of the 86 metabolites that were measured in the sample.

### Statistical analysis.

All statistical analyses were performed using GraphPad Prism software, version 7.02. The specific statistical tests used are described in the figure legends.
