# Supplementary material for: Disruption of Glycolysis by Nutritional Immunity Activates a Two-Component System That Coordinates a Metabolic and Antihost Response by Staphylococcus aureus
Source: mBio. 2019 Aug 6;10(4):e01321-19. doi: 10.1128/mBio.01321-19 (PMC6686040; doi:10.1128/mBio.01321-19)
Supplement: TABLE S1 [file mBio.01321-19-st001.docx]

**Table S1. Primers used in this study.**

| **Name** | **Sequence** |
| --- | --- |
| P2*mgrA* 5’ PstI | AAACTGCAGCGTCCCCTTTTAAAGCAATG |
| P2*mgrA* 3’ KpnI | AAGGTACCAATAAGAATATCCATAATTAACGGATTTTTGG |
| *mntC* promoter BamHI F | AGTCGGATCCTACTTTCACCTCACATACATTG |
| *mntC* promoter KpnI R | AGTCGGTACCAACGTTTATACCTCCTAATTAAAAG |
