# Supplementary material for: Disruption of Glycolysis by Nutritional Immunity Activates a Two-Component System That Coordinates a Metabolic and Antihost Response by Staphylococcus aureus
Source: mBio. 2019 Aug 6;10(4):e01321-19. doi: 10.1128/mBio.01321-19 (PMC6686040; doi:10.1128/mBio.01321-19)
Supplement: TABLE S2 [file mBio.01321-19-st002.docx]

**Table S2. *Staphylococcus aureus* strains used in this study.**

| **Bacterial Strains** | **Genotype** | **Source** |
| --- | --- | --- |
| Newman | wild type | [[8](#_ENREF_8)] |
| Newman Δ*arlR* | *arlR::erm* | [[8](#_ENREF_8)] |
| Newman Δ*mntC*Δ*mntH* | Δ*mntC*Δ*mntH* | [[8](#_ENREF_8)] |
| Newman Δ*arlR*Δ*mntC*Δ*mntH* | *arlR::erm*Δ*mntC*Δ*mntH* | [[8](#_ENREF_8)] |
| Newman pEmpty | wild type carrying pAH5::empty | [[63](#_ENREF_63)] |
| Newman Δ*arlR* pEmpty | *arlR::erm* carrying pAH5::empty | This study |
| Newman Δ*mntC*Δ*mntH* pEmpty | Δ*mntC*Δ*mntH* carrying pAH5::empty | This study |
| Newman Δ*arlR*Δ*mntC*Δ*mntH* pEmpty | *arlR::erm*Δ*mntC*Δ*mntH* carrying pAH5::empty | This study |
| Newman pAH5::P*mgrA* | wild type carrying pAH5::P*mgrA* | This study |
| Newman Δ*arlR* pAH5::P*mgrA* | *arlR::erm* carrying pAH5::P*mgrA* | This study |
| Newman Δ*mntC*Δ*mntH* pAH5::P*mgrA* | Δ*mntC*Δ*mntH* carrying pAH5::P*mgrA* | This study |
| Newman Δ*arlR*Δ*mntC*Δ*mntH* pAH5::P*mgrA* | *arlR::erm*Δ*mntC*Δ*mntH* carrying pAH5::P*mgrA* | This study |
| Newman pAH5::P*mntC* | wild type carrying pAH5::P*mntC* | This study |
| Newman Δ*arlR* pAH5::P*mntC* | *arlR::erm* carrying pAH5::P*mntC* | This study |
| Newman Δ*mntC*Δ*mntH* pAH5::P*mntC* | Δ*mntC*Δ*mntH* carrying pAH5::P*mntC* | This study |
| Newman Δ*arlR*Δ*mntC*Δ*mntH* pAH5::P*mntC* | *arlR::erm*Δ*mntC*Δ*mntH* carrying pAH5::P*mntC* | This study |
| Newman Δ*gpmA* pEmpty | *gpmA::erm* carrying pAH5::empty | This study |
| Newman Δ*gpmI* pEmpty | *gpmI::erm* carrying pAH5::empty | This study |
| Newman Δ*gpmA* pAH5::P*mgrA* | *gpmA::erm* carrying pAH5::P*mgrA* | This study |
| Newman Δ*gpmI* pAH5::P*mgrA* | *gpmI::erm* carrying pAH5::P*mgrA* | This study |
| USA300 (JE2) | wild type | NTML  (ID: NR-46543) |
| USA300 (JE2) Δ*arlR* | *arlR::erm* | NTML  (ID: NR-48226) |
| USA300 (JE2) pEmpty | wild type carrying pAH5::empty | This study |
| USA300 (JE2) Δ*arlR* pEmpty | *arlR::erm* carrying pAH5::empty | This study |
| USA300 (JE2) pAH5::P*mgrA* | wild type carrying pAH5::P*mgrA* | This study |
| USA300 (JE2) Δ*arlR* pAH5::P*mgrA* | *arlR::erm* carrying pAH5::P*mgrA* | This study |
| USA300 (JE2) pAH5::P*mntC* | wild type carrying pAH5::P*mntC* | This study |
| USA300 (JE2) Δ*arlR* pAH5::P*mntC* | *arlR::erm* carrying pAH5::P*mntC* | This study |
| USA300 (JE2) pXen1 | wild type carrying pXen1 | This study |
| USA300 (JE2) Δ*arlR* pXen1 | *arlR::erm* carrying pXen1 | This study |
| USA300 (JE2) pXen1::P*lukE* | wild type carrying pXen1::P*luxE* | This study |
| USA300 (JE2) Δ*arlR* pXen1::P*luxE* | *arlR::erm* carrying pXen1::P*luxE* | This study |
| USA300 (JE2) pXen1::P*luxS* | wild type carrying pXen1::P*luxS* | This study |
| USA300 (JE2) Δ*arlR* pXen1::P*luxS* | *arlR::erm* carrying pXen1::P*luxS* | This study |

NTML = Nebraska Trasnposon Mutant Library
