# Supplementary material for: Disruption of Glycolysis by Nutritional Immunity Activates a Two-Component System That Coordinates a Metabolic and Antihost Response by Staphylococcus aureus
Source: mBio. 2019 Aug 6;10(4):e01321-19. doi: 10.1128/mBio.01321-19 (PMC6686040; doi:10.1128/mBio.01321-19)
Supplement: TABLE S3 [file mBio.01321-19-st003.docx]

**Table S3. Plasmids used in this study.**

| **Name** | **Description** | **Source** |
| --- | --- | --- |
| pAH5 | YFP reporter plasmid | [[63](#_ENREF_63)] |
| pEmpty | pAH5 lacking a promoter for the expression of YFP | [[98](#_ENREF_98)] |
| pAH5::P*mgrA* | Plasmid for *mgrA* promoter 2-dependent YFP expression | This study |
| pAH5::P*mntC* | Plasmid for *mntC* promoter-dependent YFP expression | This study |
| pXen1 | pXen1 lacking a promoter for the expression of Lux | [[104](#_ENREF_104)] |
| pXen1::P*lukE* | Plasmid for *lukE* promoter-dependent Lux expression | [[105](#_ENREF_105)] |
| pXen1::P*lukS* | Plasmid for *lukS* promoter-dependent Lux expression | [[106](#_ENREF_106)] |
| pS100A8 | CP expression construct | [[38](#_ENREF_38)] |
| pS100A9 | CP expression construct | [[38](#_ENREF_38)] |
| pS100A8 H17N, H27N | CP ∆Mn/Zn site expression construct | [[38](#_ENREF_38)] |
| pS100A9 H91N, H95N | CP ∆Mn/Zn site expression construct | [[38](#_ENREF_38)] |
| pS100A8 H83N, H87N | CP ∆Zn site expression construct | [[38](#_ENREF_38)] |
| pS100A9 D30S, H20N | CP ∆Zn site expression construct | [[38](#_ENREF_38)] |
| pS100A8 H17N, H27N, H83N, H87N | CP ∆Mn/Zn and ∆Zn site expression construct | [[38](#_ENREF_38)] |
| pS100A9 H20N, D30S, H91N, H95N | CP ∆Mn/Zn and ∆Zn site expression construct | [[38](#_ENREF_38)] |
